# Quantitative evaluation of the symptoms provoked during the oral food challenge using the Anaphylaxis Scoring Aichi (ASCA)

**DOI:** 10.1186/2045-7022-3-S3-P105

**Published:** 2013-07-25

**Authors:** K Ito, N Kando, A Hino

**Affiliations:** 1 Department of Allergy, Aichi Children's Health and Medical Center, Obu, Japan; 2 Department of Pediatrics, Midori Hospital, Gifu, Japan

## Background

Quantitative evaluation of the severity of allergic symptoms is needed in clinical research to establish a standard policy for the management of food allergies. However, no appropriate symptom scoring system has been available so far. We have created an original symptom scoring sheet named the ‘Anaphylaxis Scoring Aichi (ASCA)’ to be used during an oral food challenge (OFC, EAACI 2012, Geneva). The ASCA lists and sorts subjective and objective symptoms into five organs (respiratory, skin-mucosal, gastrointestinal, psycho-neurological and cardiovascular). The organ scores are given (0 to 60 points) in accordance to the severity of each symptom. The total score was defined as the sum of the highest five organ scores (maximum 240 points) observed throughout the course of an OFC.

## Methods

We have evaluated positive OFCs to boiled egg white (n=374), raw milk (n=213) and udon noodle (for wheat) (n=136), which were performed from April 2011 to October 2012 with written informed consent and approval of the ethics committee. A total of 233 negative OFCs during the period were not included in the study. The target food was typically given every 20 minutes in an increasing dose from 0.1 to 20 – 50 g until the objective allergic symptoms were observed. The total score was then divided by the accumulated protein dose (g) to show the overall severity and threshold dose of the allergic reaction (TS/Pro). The correlation between the TS/Pro and specific IgE titers (ImmunoCAP, Phadia, Uppsala) was examined.

## Results

The total scores ≥100 points were recorded in five egg white, two milk and four wheat challenges. The distribution of the total scores was not different among the target foods, and in total, 44.9% had a total score ≤10 points, 46.9% were between 11 to 40 points, and 8.1% were ≥41 points. A significant correlation coefficient was detected between the TS/Pro and sIgE for egg white (r=0.24, p<0.001), ovomucoid (r=0.28, p<0.001), milk (r=0.23, p<0.001), wheat (r=0.36, p<0.001), and omega-5 gliadin (r=0.41, p<0.001).

## Conclusion

The ASCA enables a quantitative analysis of the results of a OFC, and specific IgE titers were shown to correlate with the TS/Pro, which represents the overall severity of the allergic reactions.

## Disclosure of interest

None declared.

